# Effects of Transglutaminase on Myofibrillar Protein Composite Gels with Addition of Non-Meat Protein Emulsion

**DOI:** 10.3390/gels9110910

**Published:** 2023-11-17

**Authors:** Mangang Wu, Qing Yin, Junjie Bian, Yuyu Xu, Chen Gu, Junying Jiao, Jingjing Yang, Yunlin Zhang

**Affiliations:** College of Food Science and Engineering, Yangzhou University, Yangzhou 225127, China; 15250740978@139.com (Q.Y.); 18762329580@163.com (J.B.); xyy15375139416@126.com (Y.X.); 15951709118@163.com (C.G.); 18961670649@163.com (J.J.); 19944577674@163.com (J.Y.); 18208703446@163.com (Y.Z.)

**Keywords:** TGase, non-meat proteins, myofibrillar protein, composite gel properties

## Abstract

The emulsions prepared by three non-meat proteins, sodium caseinate (SC), soy protein isolate (SPI) and egg white protein (EPI), were individually added to the continuous phase of myofibrillar protein (MP) sol to form MP composite gels to simulate meat products. The research aimed to investigate the effects of Transglutaminase (TGase) on the physicochemical properties, microstructure and water phase distribution of non-meat protein emulsion MP composite gels. The results of this study revealed that TGase played a crucial role in forming a tight gel network structure in the composite gels. This enhanced their ability to retain water and improved their overall gel strength. Additionally, TGase increased the gel formation temperature of myofibrillar proteins. Electrophoresis analysis showed that when catalyzed by TGase, there was a lighter band compared to those not catalyzed by TGase. This indicated that the addition of TGase facilitated cross-linking interactions between meat proteins and non-meat proteins in the composite gels. Furthermore, microscopy observations demonstrated that composite gels treated with TGase exhibited a more uniform microstructure. This could be attributed to an acceleration in relaxation time T2. The uniform network structure restricted the movement of water molecules in the gel matrix, thereby improving its water-holding capacity. Overall, these findings highlight how incorporating non-meat proteins into myofibrillar systems can be effectively achieved through enzymatic treatment with TGase. Such modifications not only enhanced important functional properties but also contributed towards developing alternative meat products with improved texture and moisture retention abilities.

## 1. Introduction

Myofibrillar protein plays an important role in the processing of meat products. Under heating conditions, a certain concentration of myofibrillar protein will undergo a series of physical and chemical reactions to form gels [1]. Thermally induced protein-protein association is recognized as a key molecular event in the transition of a viscous myofibrillar protein sol to a 3-dimensional gel matrix during meat processing [2]. The formed protein gels are important because they contribute greatly to meat binding, water entrapment, and fat immobilization in processed meat products. The gel property of myofibrillar protein is an important index for evaluating the quality of meat products, and how to improve this property is an important issue currently facing. Vegetable oil pre-emulsions mixed into MP sols substantially improved dynamic rheological properties during the sol to gel transformation, for set (cooled) gels, the incorporation of MP-coated, small oil droplets gave rise to the greatest gel reinforcement, demonstrating the stabilizing role of protein interactions at the interface and it is potential and feasibility to modify the textural properties of comminuted meat products by means of the inclusion of small-particle-size vegetable oil emulsions stabilized by MP [3].

The functional characteristics of myofibrillar protein are reflected in its ability to form a three-dimensional gel network with viscoelasticity, which interacts with water to form a stable protein film on the surface of fat globules in an emulsion system. These functional characteristics are closely related to the taste and appearance of meat products. The quality of a single gel meat product is unstable, and many consumers prefer low-fat meat products, so non-meat protein is often used as a fat substitute to add into meat products, while improving the quality of meat products. Common non-meat proteins are soy protein isolate, sodium caseinate and egg white protein, etc. Soy protein isolate (SPI) is widely used in meat products as a binder to improve yield, as a gelling agent to improve texture and as a meat substitute to decrease formulation costs, the structure–function relationship of SPI is considered very important to the quality of meat products [4]. Caseins, representing about 80% of the protein content in bovine milk, are isolated from milk by acid or by rennet precipitation. The acid, or isoelectric, precipitation is performed at pH 4.6, caseins are flexible and heat stable proteins [5]. Egg white is rich in ovalbumin (about 54%), ovotransferrin (about 12%), ovomucoid (about 11%), and lysozyme (about 3.4%) [6]. Its gelation is a complex process involving protein denaturation, aggregation and formation of gel network [7]. The gel characteristics of egg white mainly depend on the medium conditions such as pH, ionic strength and type of salts [8]. Another method that can effectively improve the quality of meat products is to modify the protein structure by adding enzymes to catalyze protein interactions.

TGase is one of the most widely used enzymes in meat products. It is often used to catalyze the reaction between proteins to improve the structure of the protein [9]. TGase can significantly improve the physical properties of meat products such as elasticity and hardness [10]. Adding TGase in the production of recombinant meat products can bind minced meat and cross-link different types of non-meat proteins (sodium caseinate, egg white protein, soy protein isolate) to meat proteins to form covalent and molecular bonds, to achieve the purpose of improving gelation and improve the nutritional value of protein while improving the taste and flavor of meat products [11]. Nonaka et al. [12] improved the gelation properties of soy protein isolate by adding TGase. The experimental results showed that the gel strength of soy protein isolate increased with the increase of the measured TGase concentration, the cohesion reached its maximum when the TGase was added at a concentration of 1 μg/g. Ramirezsuarez et al. [13] used TGase in a mixed system of myofibrillar protein and soy protein, and experiments found that the addition of TGase had a significant improvement on the elasticity of myofibrillar composite gel. Other studies have found that TGases can improve the gel network structure of myofibrillar protein, thereby achieving the effect of improving the water holding capacity of the gel [14].

Considerable progress achieved over the past three decades indicates the feasibility and promise to produce nutritionally improved comminuted meats with reduced health concerns through the incorporation of vegetable oil pre-emulsions to partially replace animal fat [15]. In this paper, non-meat proteins (sodium caseinate, soy protein isolate, egg white protein) and vegetable oil were pre-emulsified and added to a myofibrillar protein sol, and heated to form a composite gel. Taking the myofibrillar protein non-meat protein composite gel system as the research object, the effects of the addition of TGase on the protein composite gel system were mainly studied.

## 2. Results and Discussion

### 2.1. Effects of TGase on Gel Strength and Water Holding Capacity of the Composite Gels

**Figure 1:**
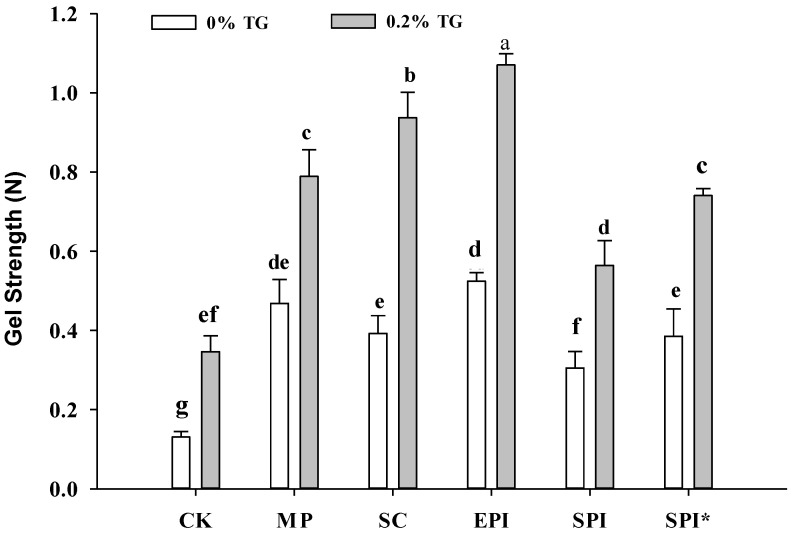
Effect of TGase on the gel strength of different non-meat protein emulsion composite gels. Note: CK: a pure MP gel without the addition of emulsion. MP: composite gel (2% protein and 10% oil) with MP emulsion (1% MP and 20% oil) added to MP suspension. SC: composite gel (2% protein and 10% oil) with SC emulsion (1% SC and 20% oil) added to MP suspension. EPI: composite gel (2% protein and 10% oil) with EPI emulsion (1% EPI and 20% oil) added to MP suspension. SPI: composite gel (2% protein and 10% oil) with SPI emulsion (1% SPI and 20% oil) added to MP suspension. SPI* Indicates pre-heating SPI. Values of different groups with different lowercase letters (a–g) are significantly different at *p* < 0.05. Error bars were standard errors of the mean (*n* = 3).

To elucidate the role of different non-meat protein emulsion and TGase on the MP gel properties, gels prepared with various non-meat protein emulsion and TGase were evaluated by a penetration test and water holding capacity (WHC) research. As shown in Figure 1. The gel strength of the composite gel formed by adding non-meat protein (SC, EPI, SPI, and SPI* group) or meat protein emulsion (MP group) was significantly stronger (*p* < 0.05) compared with the control group (CK, no emulsion added). Xiong, Blanchard, & Means [16] found that fat (or oil) through protein emulsification can form protein film wrapped around fat (or oil) particles. Such emulsion particles can participate in the formation of gel network structure as a copolymer, thereby enhancing gel strength of the gel system. Different protein membranes will interact with continuous phase proteins, and we can see that among the composite gels without TGase added, the gel strength of the EPI group was significantly higher than the other components, which was consistent with the research report by Chang-Lee [17]. The gel strength of SPI-emulsion composite gel was lower than that of the other composite gel groups (*p* < 0.05). The possible reason is that SPI’s ability to competitively emulsify oil is weaker than SC, EPI, and preheated SPI*. Myofibrillar protein (MP) was originally used to form gels in meat products. The gel strength of the SPI* group was significantly higher than that of the no preheated SPI group (*p* < 0.05), indicating that the preheat treated SPI can improve the MP composite gel structure and enhance the gel strength.

As shown in Figure 1, The addition of TGase can significantly improve the gel strength of corresponding MP-emulsion composite gel (*p* < 0.05). Sakamoto, Kumazawa, & Motoki [18] studied the content of ε- (γ-glutamyl) -lysine in the TG enzymatic reaction, and found that the breaking strength of caseinate and gelatin increased sharply with increase in TGase concentration. This shows that the TGase catalyzes the interaction between proteins, and the Gln-Lys bond formed in the space structure enhances the mechanical properties of the protein gel network, which is beneficial to the stability of the gel network structure, and thus improveing the gel strength of MP gel. In our experiments, 0.2% TG was individually added in the gel samples. Among the MP-emulsion composite gels with TGase added, the gel strength of the EPI group was significantly higher than the other gel samples, and the SC group was significantly higher than the MP group (*p* < 0.05). Compared with the gel strength of SC group with TGase not added was lower than that of MP group, the possible reason is that the TGase can catalyze the cross-linking of the same protein or different proteins, and SC has been shown to be a good substrate for TGase [19]. The addition of SC emulsion to myofibrillar protein is catalyzed by TGase enzyme to form a more powerful gel structure.

**Figure 2:**
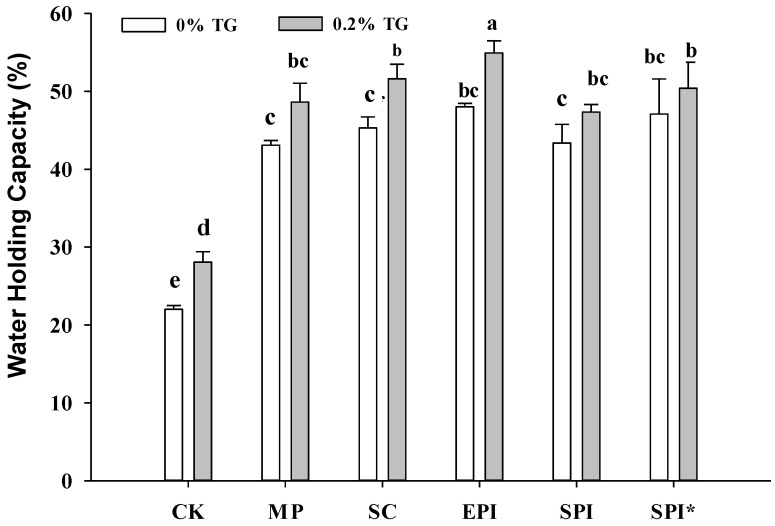
Effect of 0% TGase and 0.2% TGase on water holding capacity of composite gels. Note: CK: a pure MP gel without the addition of emulsion. MP: composite gel (2% protein and 10% oil) with MP emulsion (1% MP and 20% oil) added to MP suspension. SC: composite gel (2% protein and 10% oil) with SC emulsion (1% SC and 20% oil) added to MP suspension. EPI: composite gel (2% protein and 10% oil) with EPI emulsion (1% EPI and 20% oil) added to MP suspension. SPI: composite gel (2% protein and 10% oil) with SPI emulsion (1% SPI and 20% oil) added to MP suspension. SPI* Indicates pre-heating SPI. Values of different groups with different lowercase letters (a–e) are significantly different at *p* < 0.05. Error bars were standard errors of the mean (*n* = 3).

The effect of TGase on the water holding capacity (WHC) of MP composite gel is shown in Figure 2. Water holding capacity is one of the important indicators reflecting gel performance, which characterizes the water retention capacity of the gel network structure [20,21]. In general, protein emulsion added significantly contributed to the WHC of MP composite gel much higher than CK group. As shown from Figure 2, EPI group with TG enzymes has the best water retention performance. However, non-meat protein (SC, EPI, SPI, and SPI*) groups without TGase have similar WHC effect with MP-emulsion group (*p* > 0.05). While the addition of TGase could significantly improve the water retention of the composite gel (*p* < 0.05). After emulsion protein are catalyzed by TGase, more water molecules are distributed as bound water in the gel network, and the free water content reduced. After enzyme catalyzed, water binds more closely to non-meat protein and meat protein composite gels. Some researchers have confirmed that the TGase catalyzed the protein to enhance the water retention of the gel by catalyzing the formation of Gln-Lys bonds, mainly because the formation of cross-linking bonds made the myofibrillar protein gel spatial network structure more stable, thereby improving its ability to contain and restrain water. Luo [22] proved that an increasement in TGase concentration during incubation from 0 to 5 U/g led to an approximately 20% enhancement in the WHC of the emulsion gels. Wang also suggested that the structure and strength were the primary factors determining the WHC of the gel, just as strong and uniform structures tend to “bind” water to a greater extent [23].

### 2.2. Rheological Properties of MP Composite Gelling Solutions

**Figure 3:**
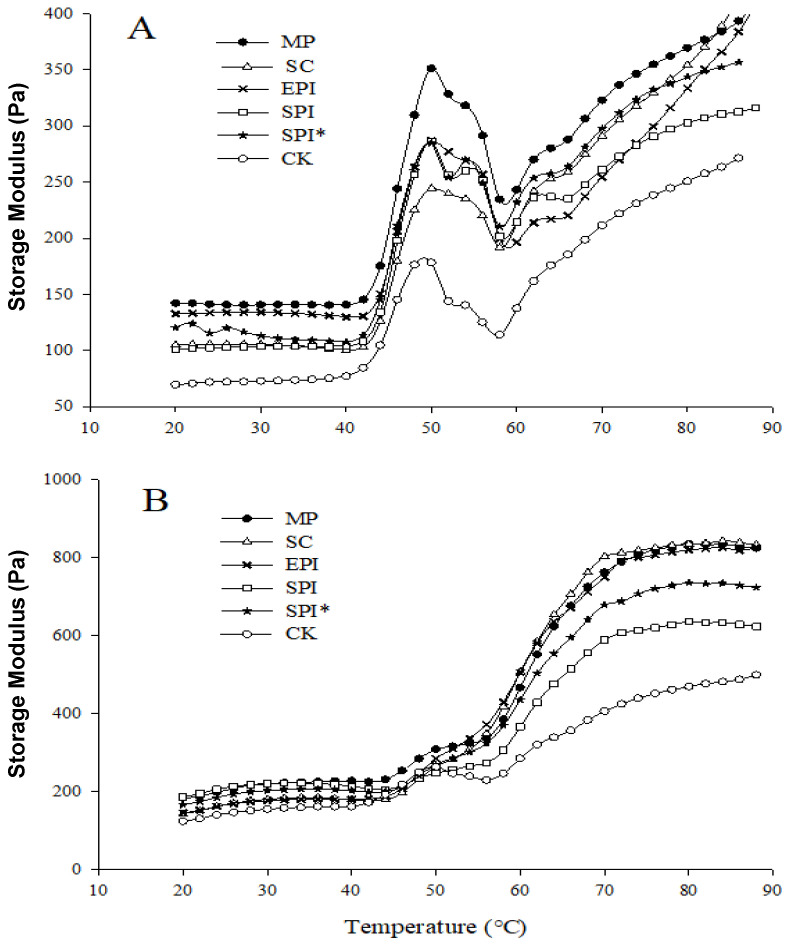
(**A**) Effect of 0% TGase on storage modulus of composite gels; (**B**) Effect of 0.2% TGase on storage modulus of composite gels. Note: CK: a pure MP gel without the addition of emulsion. MP: composite gel (2% protein and 10% oil) with MP emulsion (1% MP and 20% oil) added to MP suspension. SC: composite gel (2% protein and 10% oil) with SC emulsion (1% SC and 20% oil) added to MP suspension. EPI: composite gel (2% protein and 10% oil) with EPI emulsion (1% EPI and 20% oil) added to MP suspension. SPI: composite gel (2% protein and 10% oil) with SPI emulsion (1% SPI and 20% oil) added to MP suspension. SPI* Indicates pre-heating SPI.

Protein rheology is an important indicator of the structural characteristics of proteins. Storage Modulus (G′) reflects the elastic (or solid-like) properties of the emulsion gel and is generally used as an indicator of gel rheological property [24]. Generally speaking, the higher the G′, the better the rheological properties of the protein gel formed by myofibrillar protein. The effect of TGase on the rheology of myofibrillar composite gel is shown in Figure 3. The G′ of all the samples gradually increased at the beginning in the process of temperature increase from Figure 3A, and gel formation started at about 43 °C. It also can be seen from Figure 3B that the G′ of each component added with TGase increases rapidly between 55 °C and 65 °C, Gln-Lys cross-linking between myofibrillar protein and non-meat protein were catalyzed by TGase during this period. With increasing temperature and more complex gel networks were formed, leading to an increase in G′. Compared to the groups without the addition of TGase, the time that TGase-treated myofibrillar protein started to form gel was earlier, and the temperature required for crosslinked proteins to form elastic structures was lower. It indicates the interconnected proteins can form a measurable elastic protein gel network structure at a lower temperature. And the G′ of each component was approximately twice the G′ of each component without added TGase.

After adding TGase to different non-meat protein composite gels, the rheological curve began to show a large gap after about 55 °C. It can be seen that at the end of the temperature of 80 °C, the elastic modulus of the SPI composite gel was relatively lower without the addition of TGase or the addition of TGase, which may be related to the poor emulsification characteristics of SPI emulsion. The G′ of the pre-heated SPI* was higher than the G′ of the un-preheated SPI. This may be because the pre-heat treatment made the SPI exposed more reactive groups, and TGase catalysis strengthened the cross-linking between the SPI and MP, so the rheological properties were enhanced [13]. At the same time, the G′ of the EPI group were not significantly different from those of the MP and SC group when 0.2%TGase is added, while the G′ of the MP group was greater than the G′ of the SC group and the EPI group when no TGase is added. The possible reason was that the catalysis of TGase promotes the interaction between myofibrillar protein and non-meat protein Effect, so that the rheological properties of these two non-meat protein composite gels were enhanced. Ouyang, Y [25] proved that Compared with MP/WG system without TGase added, G′ of the system with TGase added increased more rapidly. This may be due to the formation of stable protein crosslinking structures catalyzed by TGase. This structure is not easy to be destroyed at high temperature, resulting in an increase in the elastic modulus of the gel [26].

### 2.3. Analysis of Protein Not Involved in Shangqing Liquid

**Figure 4:**
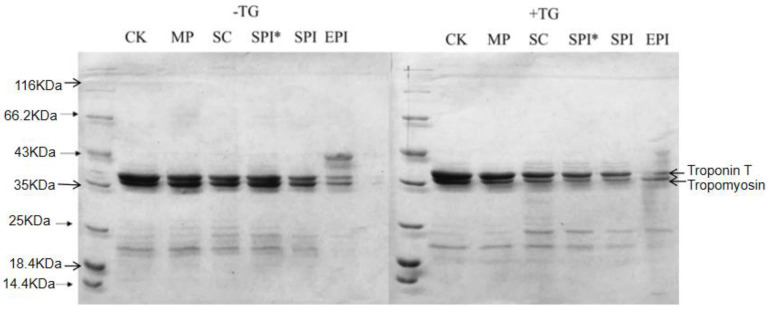
SDS-PAGE of the supernatants of TGase—induced centrifuged myofibrillar protein composite gels. Note: CK: a pure MP gel without the addition of emulsion. MP: composite gel (2% protein and 10% oil) with MP emulsion (1% MP and 20% oil) added to MP suspension. SC: composite gel (2% protein and 10% oil) with SC emulsion (1% SC and 20% oil) added to MP suspension. EPI: composite gel (2% protein and 10% oil) with EPI emulsion (1% EPI and 20% oil) added to MP suspension. SPI: composite gel (2% protein and 10% oil) with SPI emulsion (1% SPI and 20% oil) added to MP suspension. SPI* Indicates pre-heating SPI.

The electrophoretic analysis of the supernatant of centrifuged MP-emulsion composite gels was performed to examine the protein bands that remained extractable. As shown in Figure 4, supernatants from all gel samples consisted myofibrillar components: troponion T, tropomyosin, and some myosin light chain (MLC). The most obvious protein bands were troponin T and tropomyosin. Quantitative analysis using ImageJ showed that the protein content of CK, MP, SC, SPI*, SPI, and EPI composite gel supernatants without TGases were 12.68%, 11.41%, 8.18%, 10.81%, 6.47% and 4.56% respectively; the protein content of CK, MP, SC, SPI*, SPI, and EPI composite gel supernatants with TGase added were respectively 12.44%, 9.37%, 8.16%, 6.22%, 5.35%, 4.31%.

The results showed that the gray value of the protein bands of each composite gel decreased after the addition of TGase. The possible reason was that the addition of TGase catalyzed the reaction between troponin T, tropomyosin (especially) with protein in the emulsion system. so, the content of the two protein bands especially tropo-myosin in the supernatant decreased and the color of the protein bands changed lighter. Sun & Arntfield [27] reported the effect of TGase addition on the protein composition of pea protein/myofibrillar composite gel. Compared with the electrophoresis protein bands, it was found that the gel band catalyzed by TGase was lower than that in the blank group, and the myosin heavy chain and tropomyosin disappeared. The reason for this difference may be that TGases catalyzed the cross-linking of proteins to form macromolecular polymers, which were difficult to dissolve and difficult to enter the separation gel. Compared with unheated SPI, there was no significant difference in electrophoretic bands between preheated SPI and unheated SPI. Ramirezsuarez, & Xiong [13] also obtained consistent research results, that preheating SPI delayed the reappearance of MHC and actin but did not affect the overall electro-phoretic bands. This indicated that the presence of partially denatured soy protein made it difficult for the modified globulin to react with the TGase during the reverse protein crosslinking reaction.

### 2.4. Effects of TGase on the Microstructure of Composite Gels (ESEM)

**Figure 5:**
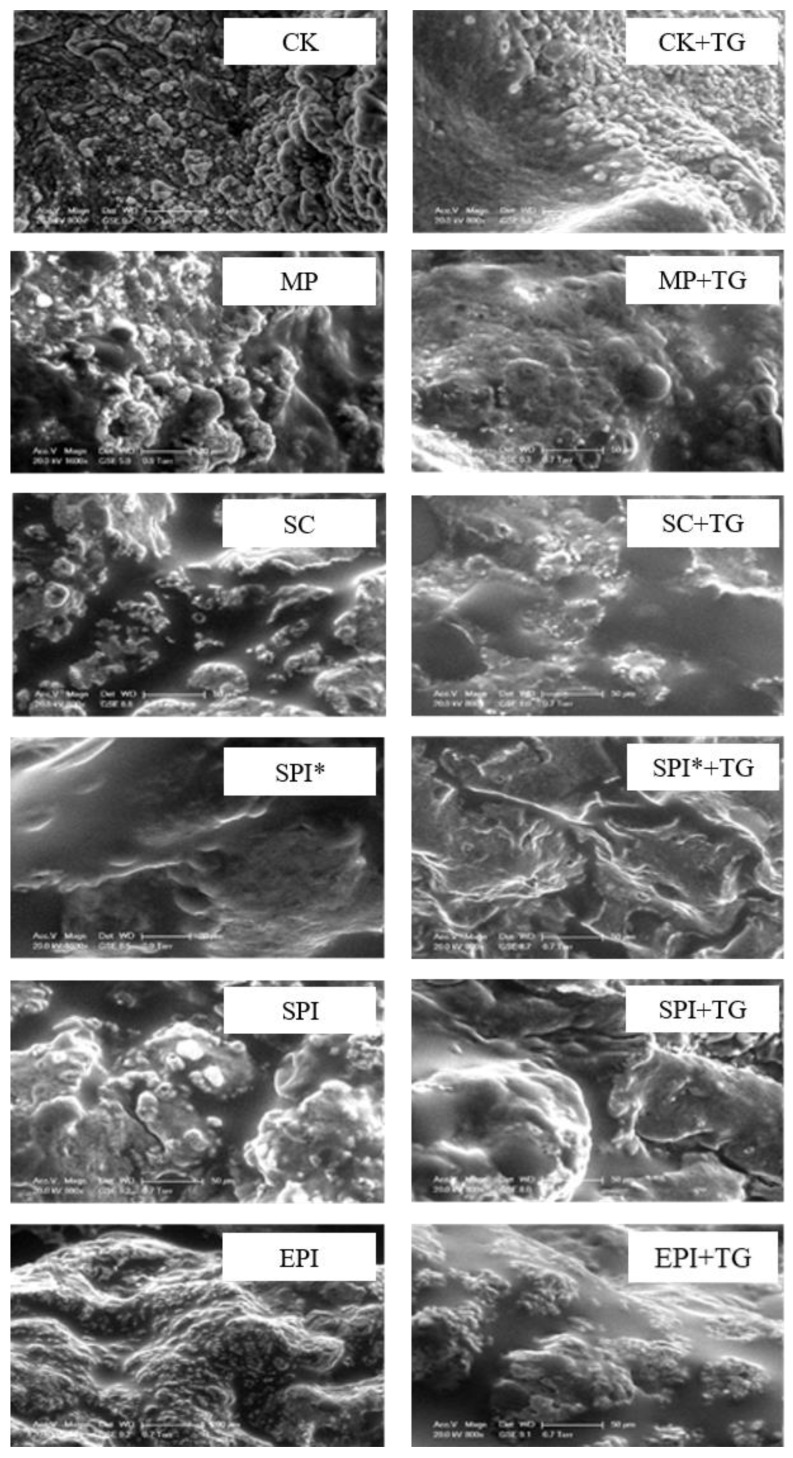
ESEM microscope of composite gels induced by TGase. Note: CK: a pure MP gel without the addition of emulsion. MP: composite gel (2% protein and 10% oil) with MP emulsion (1% MP and 20% oil) added to MP suspension. SC: composite gel (2% protein and 10% oil) with SC emulsion (1% SC and 20% oil) added to MP suspension. EPI: composite gel (2% protein and 10% oil) with EPI emulsion (1% EPI and 20% oil) added to MP suspension. SPI: composite gel (2% protein and 10% oil) with SPI emulsion (1% SPI and 20% oil) added to MP suspension. SPI* Indicates pre-heating SPI.

In order to observe the internal microstructure of the composite gel, the microstructure of the myofibrillar composite gel without TGase and TGase added is shown in Figure 5. The blank group had a rough tissue structure and formed a granular gel structure. After adding non-meat protein emulsion, some oil particles were distributed on the surface of myofibrillar protein, which indicated that myofibrillar protein emulsified coagulation there is not only protein-protein interactions but also protein-oil interactions in the gel. After the addition of TGase, the rough microstructure of myofibrillar protein gel was improved, and the microscopic surface became smooth and consistent. Chin, Go, & Xiong [28] Studies have compared the effects of TGase on the myofibrillar proteins different ionic strengths. The results showed that the TGase catalyzed the cross-linking between proteins to reduce the gaps in the myofibrillar protein network structure and transform the aggregated gel structure into a fine-chain gel structure. At the same time, Trespalacios & Pla [29] proved that under 0.1 M NaCl condition, chicken gel without TGase had larger pores, but after adding TGase the gel formed a more complex and consistent network structure.

### 2.5. Low-Field NMR Analysis of Water Phase Distribution in Gel

**Figure 6:**
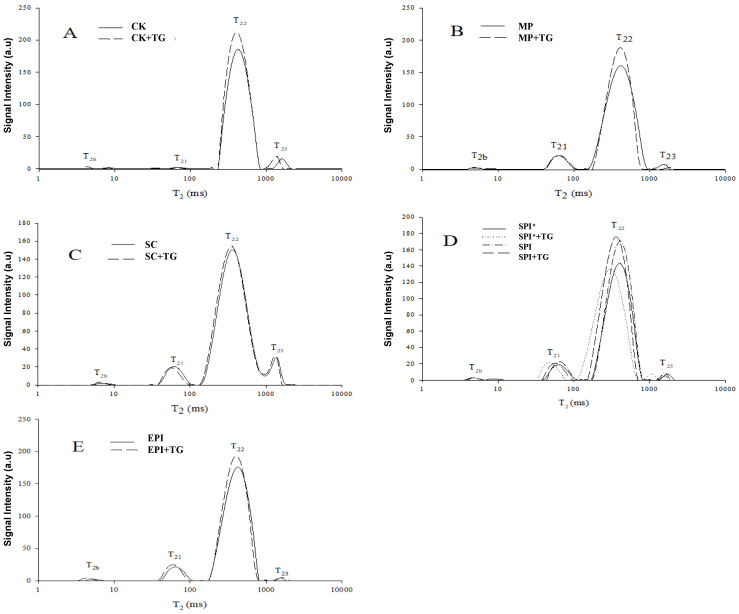
Changes in distributions of T2 relaxation times of myofibrillar composited gels treated by TGase. Notes: (**A**): T2 relaxation time changes of CK, CK + TG; (**B**): T2 relaxation time changes of MP, MP + TG; (**C**): T2 relaxation times of SC, SC + TG; (**D**): T2 relaxation time changes of SPI* + TG, SPI*, SPI and SPI + TG; (**E**): EPI, T2 relaxation time of EPI + TG; * Indicates pre-heating of SPI.

NMR technology can measure the distribution of different water phases without destroying the gel structure, so it is used to analyze the effect of TGase on the moisture distribution of myofibrillar composite gel. The low-field NMR relaxation time T2 distribution of the different non-meat protein composite gels after the fitting is four peaks, as shown in Figure 6. Related studies have confirmed that T2b partly reflects water closely related to macromolecules [30], T21 partly reflects water or oil protons that are tightly bound to proteins in the gel structure, and T22 part is considered to be non-flowing water and fat in the gel structure [31,32,33]. Because the relaxation times T2 of the second and third peaks are similar, the second and third peaks can be classified as difficult to flow water. The fourth peak appears to be due to free water and fat that are precipitated in the gel protein network structure. Hinrichs et al. [34] also detected four peaks were when whey protein gel was measured by NMR technology, and they corresponded to different states of four kinds of water, namely immovable water, weakly movable water, moderately movable water and movable water, moderately movable water accounted for the largest proportion. Table 1 shows the Changes in distributions of T2b, T21, T22, T23 relaxation times of myofibrillar composited gels treated by TGase. Table 2 shows the Changes in distributions of T21, T22, T23, T24 proportion of peak areas of myofibrillar composited gels treated by TGase.

The change in T_2_ relaxation time of CK, CK + TG is shown in Figure 6A. The relaxation times T_2_ corresponding to the four peaks of CK were: T_2b_: 4.03~9.32 ms, T_21_: 28.48~100 ms, T_22_: 231.01~811.13 ms, T_23_: 1072.27~1873.82 ms. The change in T_2_ relaxation time of MP, MP + TG’s T_2_ is shown in Figure 6B. The relaxation times T2 corresponding to the four peaks were: T_2b_: 4.04~12.33 ms, T_21_: 43.28~100 ms, T_22_: 174.75~811.13 ms, T_23_: 1232.85~2154.43 ms. T_2_ relaxation time changes of SC, SC + TG is shown in Figure 6C. The relaxation times T_2_ corresponding to the four peaks were: T_2b_: 5.34~9.32 ms, T_21_: 43.28~100 ms, T_22_: 151.999~1072.27 ms, T_23_: 932.630~1629.75 ms. The appearance of the fourth peak indicated that moderately mobile water and free water or fat were transformed during the gel formation process, and part of the combined water or fat was transformed into juice to precipitate the protein gel network. The reason may be that during the heating process of the heat-induced gel, the interaction between SC and MP and between oil molecules formed an emulsified gel, the conformational change of MP precipitates a part of the moisture absorbed in the protein during the homogenization process or the protein-encapsulated fat molecules, resulting in the loss of juice and the formation of the fourth peak. The second peak and the third peak of the SC/MP composite gel were connected, which may be caused by the interference caused by the fat signal in this figure. The change of T2 relaxation time of SPI*, SPI* + TG, SPI, and SPI + TG is shown in Figure 6D. The relaxation times T_2_ corresponding to the four peaks were: T_2b_: 4.03~12.33 ms, T_21_: 32.74~114.97 ms, T_22_: 114.97~705.48 ms, T_23_: 811.13~1873.82 ms. The change in T_2_ relaxation time of EPI, EPI + TG’s T_2_ relaxation time is shown in 6E. The relaxation times T2 corresponding to the four peaks were: T_2b_: 3.51~8.11 ms, T_21_: 43.28~100 ms, T_22_: 200.92~705.48 ms, and T_23_: 1232.85~1629.75 ms.

Comparing and analyzing the different non-meat protein composite gels, the relaxation time of the T_22_ peak (peak with the largest peak area) was significantly reduced (*p* < 0.05) when compared with the blank group, and the peak area percentage was significantly reduced (*p* < 0.05), indicating that the myofibrillar gel network structure was more compact after adding the emulsion, thereby limiting the movement of water protons. Compared with the myofibrillar gel, the T_21_ peak area percentage of each group was significantly higher, indicating that the addition of non-meat protein formed a composite gel, and the content of weakly moving water in the gel would increase significantly. The T_2_ relaxation time of preheated SPI and unpreheated SPI composite gels showed that the T_2_ relaxation time of the T_2b_ peak of preheated SPI composite gels was significantly smaller than (*p* < 0.05) the T_2_ relaxation time of unpreheated SPI composite gels This means that after pre-heating the SPI, SPI exposes more active sites to interact with myofibrillar protein to form a more stable network structure, so that the water inside the protein is well stored inside [35].

The T_2b_ and T_21_ peak area percentages in the blank group were lower, indicating that there was less bound water and more mobile water in the blank group. After adding TGase to the blank group, the T_2_ relaxation time corresponding to the four peaks moved to the direction of rapid relaxation (*p* < 0.05), and the corresponding peak area A_2b_ increased (*p* < 0.05), indicating that the addition of TGase caused the water molecules inside the gel structure bind more tightly to myofibrillar proteins. After adding TGase, the relaxation time of different non-meat protein composite gels shifted significantly to the left. The decrease in relaxation time indicated that TGase would make water molecules bind more tightly to myofibrillar protein and reduce the fluidity of water. This phenomenon was consistent with the measurement results of water holding capacity, indicating that the addition of TGase can significantly improve the water holding capacity of the composite gel. In terms of the effect of TGase on the peak area percentage of non-meat protein composite gels, in general, the T_2b_ peak area percentage (immovable water) increased significantly after adding TGase (*p* < 0.05), and the T_22_ peak area did not change significantly.

## 3. Conclusions

The TGase catalyzed the cross-linking between proteins, and the Gln-Lys bond formed was beneficial to stabilize the gel network structure. MP with non-meat protein emulsion formed a tighter gel space network structure catalyzed by TGase, therefore improving the ability to contain and restrain water. At the same time, the gel strength of MP emulsion composite gel was improved. Rheology properties indicated TGase-treated myofibrillar protein started gelling earlier. The electrophoretic band of the supernatant protein after TGase catalysis was lighter than the gel electrophoresis band without TGase. The reason for this difference may be that TGase catalyzed the cross-linking of proteins to form macromolecular polymers, these polymers were more difficult to dissolve and did not easily enter the separation gel. The addition of TGase caused cross-linking between protein macromolecules to form a more uniform gel structure. The uniform microstructure of the gel also had a clear relationship with the relaxation time. The uniform gel structure restricted the movement of water protons, reduced the relaxation time T_2_, and improved the water retention of the myofibrillar gel. The state of water retained in the network structure was different, and water in different states was in a dynamic equilibrium and would transform each other. The addition of TGase caused the T_2_ relaxation time of the gel to move to the left, that is, to the direction of rapid relaxation, which indicated that the TGase catalyzed the cross-linking between proteins and made the myofibrillar protein bind to the water molecules inside the gel more tightly, the water mobility was reduced and T_2_ relaxation time was reduced.

## 4. Materials and Methods

### 4.1. Materials

Fresh pork longissimus muscle (pH 5.6–5.9) was purchased form local market. The raw materials were brought back to the laboratory in ice box, bones and obvious external fat were removed, and the lean meat was cut into blocks and packed in 100 g portions. The portions were placed in vacuum plastic packaged bags, evacuated, and stored at −75 °C until use. Glutamine transaminase (GT, the enzyme activity was 46.8 U/g) was purchased from Taixing Dongsheng Food Technology Co., Ltd., Taixing, Jiangsu, China. The olive oil was purchased from Sinopharm Group Chemical Reagent Co., Ltd., Beijing, China. Soy protein isolate (SPI) was purchased from Zhengzhou Chenxu Chemical Products Co., Ltd., Zhengzhou, Henan, China. Egg white protein isolate (EPI) was purchased from Suzhou Oufu Egg Industry Co., Ltd., Suzhou, Jiangsu, China. Sodium caseinate (SC) was purchased from Henan Jinyuan Biotechnology Co., Ltd., Zhengzhou, China. Model DS-1 blender (Jintan Ronghua Instrument Manufacturing Co., Ltd., Jintan, Jiangsu, China). HH-8 digital constant temperature water bath pot (Guohua Electric Appliance Co., Ltd., Danjiang, Hubei, China). Sorvall ST 16R centrifuge (Thermo Fisher Scientific, Waltham, MA, USA). high-speed homogenizer (IKA T18, IKA-Werke GmbH & Co. KG, Staufen, Germany). TA-XT Plus texture analyzer (Food Technology Corporation, London, UK). AR1000 rheometer (TA Instruments, West Sussex, UK). Environmental scanning electron microscope (Thermo Fisher Scientific, Waltham, MA, USA). MesoMR23-060H-1 low-field nuclear magnetic resonance (LF-NMR) analyzer (Suzhou Newmax Analytical Instruments Co., Ltd., Suzhou, Jiangsu, China).

### 4.2. Extraction of Myofibrillar Protein (MP)

MP from the fresh lean pork was acquired on previously described methods [36]. The muscle was cut and weighed into a beaker after thawed. 4-fold volume of phosphate buffer (0.10 mol/L NaCl, 10 mmol/L sodium phosphate, 2 mmol/L MgCl_2_, 1 mmol/L EGTA, pH 7.0) and minced muscle were homogenized with a Model DS-1 blender (Jintan Ronghua Instrument Manufacturing Co., Ltd., Jintan, Jiangsu, China) at maximum speed for 60 s. After centrifuging 15 min at 2000× *g*, the obtained meat slurry was centrifuged with 2000× *g*, the precipitate was retained, and the crude MP were precipitated, chopped and centrifuged 3 times with the above buffer, the pulp was washed twice with 0.10 mol/L NaCl solution and centrifuged again under the same conditions. The obtained MP was preserved in crushed ice and used within 24–48 h.

### 4.3. Preparation of Emulsions

Preheated SPI maybe can improve the property of soy protein. So, the experiment chose two kinds of SPI, we defined preheated SPI as SPI*. Myofibrillar protein (MP), soy protein isolate (SPI and SPI*), sodium caseinate (SC), and egg white protein isolation (EPI) were dissolved in 0.6 mol/L NaCl, 0.05 mol/L Na_2_HPO_4_ solution, respectively.

The protein-stabilized emulsion was individually obtained by homogenizing the (MP, SC, EPI, SPI, SPI*) protein suspension (1%) with olive oil at a ratio of 4:1 (*w*/*w*) by a high-speed homogenizer (IKA T18, IKA-Werke GmbH & Co. KG, Staufen, Germany) at 12,000 r/min for 2 min.

### 4.4. Preparation of MP-Emulsion Composite Sols and Gels

The freshly prepared emulsion was immediately introduced into the MP suspension under gentle agitation to generate a composite sol (pH 6.25) with a final concentration of 2% (*w*/*w*) protein and 10% (*w*/*w*) oil [2]. For comparison, a pure MP gel without the addition of emulsion was also prepared as a blank control (CK). Then, 0.2% (*w*/*v*) Transglutaminase (TGase) were individually added into the corresponding MP or composite sols. Afterwards, the prepared composites (5 g) sols were cautiously placed into glass vials (22 mm in diameter × 50 mm in height), and then heated from 25 °C to 70 °C in a water bath. After heating, the gels were immediately chilled in ice water and stored at 4 °C overnight.

### 4.5. Determination of Gel Strength and Water Holding Capacity

The gel samples were placed at 25 °C and equilibrated for 30 min before measuring the gel strength of the gels. The texture profile analysis (TPA) was performed on the samples with the TA-XT Plus texture analyzer. The cylinder of P/6 was used as the probe. The penetrating force was the test strength of the gel. Each sample was tested in triplicate.

The composite gels were centrifuged at 10,000× *g* and 4 °C for 15 min. Thereafter, the supernatant was completely expelled and the precipitate was weighted. WHC (%) was defined as the weight percentage of the precipitate to the original gel [37].

### 4.6. Determination of Rheological Properties

The process of gel network formation during heating can be demonstrated using a rheometer. The viscoelastic characteristics of the MP composite sols were continuously monitored during the heat-induced gelation with a Model AR1000 rheometer (TA Instruments, West Sussex, UK) in oscillatory mode. Adding about 1.5 mL of MP composite sol (2~4 °C) between the double-layered partitions, select a 40 mm flat plate, and then tighten the two upper and lower disks with a space of 1 mm between the parallel plates, heating around the gel sample. The sample was heated from 20 °C to 80 °C at a rate of 2 °C/min. During the test, a thin layer of silicone oil needed to be added around the sample to keep the humid air to avoid evaporation of water in the gel. The sample was sinusoidally strained at an oscillation frequency of 0.631 rad/s (100 mHz) and an amplitude of 2%. The changes of the elastic modulus and the viscous modulus with the increase of temperature during the heating process were measured.

### 4.7. SDS-PAGE Analysis of Protein Involved in the Supernatant of Liquid

The composition of the protein in the supernatant obtained by the above operation was detected by SDS-PAGE electrophoresis to determine the types of proteins involved in gel formation. The acrylamide concentration of the separation gel and concentrated gel required for the experiment was 10% (*w*/*v*) and 4% (*w*/*v*) respectively. Before running electrophoresis, replenish the electrophoresis buffer to the remaining space of the electrophoresis tank. The starting voltage is 90 V for 30 min, and then adjust the voltage to 120 V for 60 min. After electrophoresis, take the glue and dye it with Coomassie brilliant blue (G250) for 60 min, and then add a decolorizing solution (10% methanol, 10% glacial acetic acid) for repeated decolorization until the background is clean and clear strips appear. Bio-Rad gel imaging system is used for image collection and analysis. All sample buffers were added with 5% (*w*/*v*) β-Mercaptoethanol to cut the disulfide bonds. The molecular weight of the standard protein ranges is 14.4 to 97.4 kDa.

### 4.8. Observation of Microstructure

The resulting gel was observed with an environmental scanning electron microscope. Cut the gel into flakes, absorb excess water from the surroundings, place the cut sample on the sampling platform for observation, and accelerate the voltage to 20.0 kV. Observe the microstructure of the gel from low to high adjustment multiples and record by adjusting the clarity the microstructure of the gel at different magnifications.

### 4.9. Composite Gel Relaxation Time Test

About 2 g of the sample was placed in a 15 mm diameter NMR tube at room temperature of 25 °C, and then placed in an analyzer. NMR T2 relaxation time test, main technical parameters: Proton resonance frequency: 22 MHz, Measurement sequence: CPMG, τ-value: 250 μs (time between 90° and 180° pulse), Repeated scanning: 32 times, Repeated interval time: 5 s, Number of echoes: 21,000, and the resulting graph is an exponential decay graph, repeated at least 4 times. The CPMG exponential decay curve was back-differentiated using MultiExp Inv Analysis software (v. 4.09, Niumag Electric Corporation, Shanghai, China) to obtain the final T2 value.

### 4.10. Statistical Analysis

The experimental data obtained were mean values after at least three replicates. SPSS 22.0 software was used for data analysis and Tukey program was used for significance analysis (*p* < 0.05).

## Figures and Tables

**Table 1:** Changes in distributions of T2b, T21, T22, T23 relaxation times of myofibrillar composited gels treated by TGase.

Combinations	CK	CK + TG	MP	MP + TG	SC	SC + TG
T_2b_ (ms)	8.23 ± 7.34 ^a^	4.32 ± 0.65 ^e^	4.96 ± 1.08 ^de^	5.75 ± 6.54 ^d^	7.29 ± 1.51 ^b^	6.65 ± 1.52 ^c^
T_21_ (ms)	69.04 ± 8.02 ^a^	35.15 ± 6.09 ^f^	63.93 ± 0.00 ^c^	66.03 ± 0.00 ^b^	62.27 ± 4.95 ^c^	58.24 ± 0.00 ^d^
T_22_ (ms)	431.68 ± 0.00 ^a^	423.07 ± 0.00 ^b^	401.51 ± 0.00 ^c^	388.92 ± 0.00 ^d^	364.04 ± 0.00 ^e^	353.29 ± 0.00 ^f^
T_23_ (ms)	1611.50 ± 122.56 ^b^	1357.80 ± 0.00 ^f^	1482.60 ± 122.56 ^e^	1818.40 ± 262.55 ^a^	1313.60 ± 106.59 ^f^	1260.70 ± 0.00 ^g^
Combinations	SPI*	SPI* + TG	SPI	SPI + TG	EPI	EPI + TG
T_2b_ (ms)	4.79 ± 1.22 ^de^	4.89 ± 1.08 ^de^	8.88 ± 1.84 ^a^	6.79 ± 4.82 ^c^	5.37 ± 1.51 ^d^	4.41 ± 0.99 ^e^
T_21_ (ms)	66.37 ± 0.00 ^b^	58.52 ± 0.00 ^d^	64.19 ± 0.00 ^bc^	49.47 ± 3.74 ^e^	64.89 ± 0.00 ^bc^	59.85 ± 0.00 ^cd^
T_22_ (ms)	393.01 ± 0.00 ^cd^	343.44 ± 0.00 ^a^	385.03 ± 0.00 ^d^	293.02 ± 0.00 ^g^	403.21 ± 19.97 ^c^	388.79 ± 0.00 ^d^
T_23_ (ms)	1552.10 ± 122.56 ^d^	1567.50 ± 122.56 ^d^	1690.10 ± 122.56 ^b^	1052.50 ± 92.71 ^h^	1517.70 ± 0.00 ^d^	1635.50 ± 140.91 ^c^

Note: a~h represents the significance of the difference in the same row of data respectively.

**Table 2:** Changes in distributions of T21, T22, T23, T24 proportion of peak areas of myofibrillar composited gels treated by TGase.

Combinations	CK	CK + TG	MP	MP + TG	SC	SC + TG
A_2b_/A (%)	0.38 ± 0.15 ^e^	0.44 ± 0.09 ^de^	0.46 ± 0.08 ^d^	0.69 ± 0.39 ^b^	0.49 ± 0.05 ^d^	0.57 ± 0.01 ^c^
A_21_/A (%)	0.66 ± 0.16 ^f^	0.31 ± 0.13 ^f^	6.71 ± 0.14 ^b^	6.78 ± 0.03 ^ab^	6.86 ± 0.26 ^d^	5.87 ± 0.07 ^e^
A_22_/A (%)	95.90 ± 0.30 ^a^	96.12 ± 0.27 ^a^	91.60 ± 0.34 ^c^	92.18 ± 0.26 ^c^	93.59 ± 0.40 ^b^	93.87 ± 0.34 ^b^
A_23_/A (%)	3.03 ± 0.30 ^a^	3.12 ± 0.25 ^a^	1.21 ± 0.13 ^c^	0.34 ± 0.15 ^d^	-	-
Combinations	SPI*	SPI* + TG	SPI	SPI + TG	EPI	EPI + TG
A_2b_/A (%)	0.66 ± 0.09 ^b^	0.59 ± 0.09 ^c^	0.44 ± 0.09 ^de^	0.09 ± 0.06 ^f^	0.60 ± 0.04 ^c^	0.83 ± 0.09 ^a^
A_21_/A (%)	7.65 ± 0.26 ^b^	6.80 ± 0.23 ^d^	7.86 ± 0.26 ^a^	7.68 ± 0.54 ^b^	7.24 ± 0.25 ^c^	7.52 ± 0.22 ^b^
A_22_/A (%)	90.38 ± 0.24 ^d^	91.96 ± 0.18 ^c^	90.21 ± 0.14 ^d^	90.73 ± 0.94 ^d^	91.56 ± 0.23 ^c^	90.88 ± 0.49 ^d^
A_23_/A (%)	1.31 ± 0.13 ^b^	0.65 ± 0.12 ^d^	1.49 ± 0.17 ^b^	1.50 ± 0.89 ^b^	0.60 ± 0.09 ^d^	0.77 ± 0.21 ^d^

Note: a~f represents the significance of the difference in the same row of data respectively.

## Data Availability

The data presented in this study are available on request from the corresponding author. The data are not publicly available due to privacy.
